# Oral *Lactobacillus crispatus* M247 Supplementation and Persistence of Vaginal Group B Streptococcus Colonization During Pregnancy

**DOI:** 10.3390/jcm15197368

**Published:** 2026-09-22

**Authors:** Mihaela Boț, Andreea Borislavschi, Ciprian Coroleucă, Radu Vlădăreanu

**Affiliations:** 1 Department of Obstetrics and Gynecology, “Carol Davila” University of Medicine and Pharmacy, 8 Eroii Sanitari Blvd., 050474 Bucharest, Romania; mihaela.bot@umfcd.ro (M.B.);; 2 Department of Obstetrics and Gynecology, Elias University Emergency Hospital, 17 Mărăști Blvd., 011461 Bucharest, Romania; 3 Department of Obstetrics and Gynecology, Prof. Dr. Panait Sirbu Clinical Hospital of Obstetrics and Gynecology, 060251 Bucharest, Romania

**Keywords:** Group B Streptococcus, pregnancy, probiotics, *Lactobacillus crispatus*, vaginal microbiota, GBS colonization

## Abstract

**Background:** Group B Streptococcus (GBS) colonization during pregnancy increases neonatal infection risk and is managed with intrapartum antibiotic prophylaxis. We evaluated whether oral probiotic supplementation was associated with reduced persistence of vaginal GBS colonization. **Methods:** This prospective, non-randomized, contemporaneous controlled intervention study included pregnant women with confirmed vaginal GBS colonization at 24–26 weeks of gestation. Participants selected either oral *Lactobacillus crispatus* M247 (20 billion colony-forming units (CFU) daily for eight weeks) or standard prenatal care. Vaginal cultures were repeated at 34–36 weeks. The primary outcome was persistent GBS colonization. Fisher’s exact test and relative risk (RR) were used. Exploratory Firth logistic regression adjusted for age, body mass index, and previous GBS colonization status; sensitivity analysis excluded unknown status. Trial registration: ISRCTN47071089, retrospectively registered. **Results:** Of 53 enrolled women (24 probiotic; 29 control), one control participant was lost to follow-up, leaving 52 for analysis. Persistent colonization was less frequent in the probiotic group than in controls (12.5% vs. 57.1%; *p* = 0.001; RR 0.219, 95% confidence interval (CI) 0.072–0.661). In the primary Firth model (n = 52; 19 events), probiotic use was associated with lower odds of persistence (adjusted odds ratio (aOR) 0.13, 95% CI 0.03–0.46; *p* = 0.001). Sensitivity analysis yielded similar results (n = 44; 15 events; aOR 0.17, 95% CI 0.03–0.63; *p* = 0.007). Self-reported adherence was complete, and no adverse events were reported among participants receiving the probiotic. **Conclusions:** Oral *L. crispatus* M247 supplementation from 24–26 weeks of gestation was associated with substantially lower persistence of vaginal GBS colonization in late pregnancy. Larger randomized controlled trials are required to confirm these findings.

## 1. Introduction

*Streptococcus agalactiae*, or Group B Streptococcus (GBS), is a Gram-positive bacterium that colonizes the gastrointestinal and genitourinary tracts and represents a significant pathogen in perinatal medicine. Maternal rectovaginal colonization is reported in approximately 10–30% of pregnant women globally, with prevalence varying according to geographic region and population characteristics [1]. Although maternal carriage is usually asymptomatic, it constitutes the principal risk factor for vertical transmission during labor and delivery. Early-onset neonatal GBS disease, typically presenting within the first days of life as sepsis, pneumonia, or meningitis, remains an important cause of neonatal morbidity and mortality worldwide despite advances in obstetric and neonatal care [2].

To mitigate the risk of neonatal infection, the American College of Obstetricians and Gynecologists (ACOG) recommends universal screening for GBS colonization between 36 0/7 and 37 6/7 weeks of gestation using a vaginal-rectal culture, followed by intrapartum antibiotic prophylaxis for women with a positive result [3].

This strategy has substantially reduced the incidence of early-onset neonatal disease; however, maternal colonization itself remains common and may vary over the course of pregnancy. Several maternal factors have been associated with an increased likelihood of GBS carriage, including alterations in the vaginal microbiota and a history of colonization in previous pregnancies. Notably, recurrence of GBS colonization in subsequent pregnancies has been reported to be significantly higher, suggesting persistence or repeated recolonization within the maternal microbial ecosystem [4].

The composition of the vaginal microbiota plays a crucial role in maintaining mucosal homeostasis and protecting against colonization by pathogenic microorganisms. In healthy reproductive-age women, the vaginal ecosystem is typically dominated by *Lactobacillus* species, which contribute to host defense through the production of lactic acid, bacteriocins, and other antimicrobial substances that maintain a low vaginal pH and inhibit pathogen growth [5]. Among these species, *Lactobacillus crispatus* has been consistently associated with a stable vaginal microbiome and favorable reproductive outcomes, including a reduced risk of bacterial vaginosis and preterm birth [6].

Despite the widespread implementation of universal screening and intrapartum antibiotic prophylaxis, maternal GBS colonization remains common during pregnancy. Current preventive strategies primarily focus on reducing neonatal transmission at delivery rather than addressing the determinants of maternal colonization. Increasing evidence suggests that the composition of the vaginal microbiota, particularly the dominance of *Lactobacillus* species, may influence susceptibility to GBS carriage. However, the potential role of microbiome-targeted interventions, such as probiotic supplementation, in modulating GBS colonization during pregnancy remains insufficiently investigated [3,5].

In this context, the aim of the present study was to evaluate whether oral supplementation with *Lactobacillus crispatus* administered during the second trimester of pregnancy would be associated with lower persistence of detectable vaginal GBS colonization at 34–36 weeks of gestation among women with confirmed GBS colonization at 24–26 weeks. Women with confirmed baseline vaginal GBS colonization who chose probiotic supplementation were compared with contemporaneous controls receiving standard antenatal care.

## 2. Materials and Methods

This prospective, non-randomized, contemporaneous controlled intervention study was conducted between March 2025 and September 2025 at the Department of Obstetrics and Gynecology, Elias University Emergency Hospital, Bucharest, Romania. The study evaluated whether oral administration of *Lactobacillus crispatus* M247 during the second trimester was associated with lower persistence of vaginal Group B Streptococcus (GBS) colonization at 34–36 weeks of gestation among women with confirmed GBS colonization at 24–26 weeks. This report was prepared in accordance with the Transparent Reporting of Evaluations with Nonrandomized Designs (TREND) statement for non-randomized evaluations of interventions (Table S1).

The study protocol was approved by the Ethics Committee of Elias University Emergency Hospital (Approval No. 1835, 21 March 2025). The study was conducted in accordance with the principles of the Declaration of Helsinki [7]. A two-stage informed consent process was applied: during first-trimester day-hospitalization assessment, participants provided written informed consent for sequential screening sample collection (performed at baseline, 24–26 weeks, and 34–36 weeks of gestation). Subsequently, patients with confirmed vaginal GBS colonization signed a secondary written informed consent for formal inclusion in the study cohort.

Pregnant women underwent routine antenatal assessment during scheduled day-hospital admissions at Elias University Emergency Hospital, as part of publicly funded care reimbursed by the Romanian National Health Insurance System. This care pathway enabled the clinical and paraclinical assessment of both the pregnant woman and the fetus. For the purposes of the present study, these investigations were supplemented with a study-specific GBS culture obtained from the lower third of the vagina at 24–26 weeks of gestation. The investigators were aware that national and international guidelines do not recommend routine GBS screening during the second trimester and recommend combined vaginal–rectal sampling for routine late-pregnancy screening. Therefore, the second-trimester vaginal culture was performed exclusively for research purposes, in accordance with the approved study protocol, and did not represent routine clinical GBS screening.

Pregnant women aged 18 years or older with a confirmed viable pregnancy and a positive vaginal GBS culture at 24–26 weeks of gestation were assessed for eligibility. Eligibility was determined at enrolment, before group allocation. Participants were recruited during the second trimester following confirmation of vaginal GBS colonization at 24–26 weeks of gestation. Inclusion and exclusion criteria are presented in Table 1.

Before enrolment and group selection, all eligible women invited to participate received standardized counselling delivered by two experienced obstetricians (M.B. and A.B.). The counselling covered the nature of probiotics; the vaginal microbiota; vaginal GBS colonization and its potential fetal and neonatal implications; current guideline recommendations, including the role of routine late-pregnancy screening and intrapartum antibiotic prophylaxis; and the standard clinical management of GBS colonization. Participants were also informed about the available evidence concerning probiotic use during pregnancy, its potential benefits and risks, the proposed but not established mechanisms through which orally administered probiotics might influence the vaginal microbiota, and the evidence that community state type I is characterized by the predominance of *Lactobacillus crispatus*. The specific hypothesis evaluated in this study and its investigational nature were clearly explained. Participants were informed that the study aimed to assess detectable vaginal GBS colonization and did not aim to modify or evaluate rectal colonization. The same information was presented to all eligible women, and group allocation was determined exclusively by each participant’s preference.

Participants were allocated to a probiotic group or a control group in a non-randomized manner according to patient preference following counselling regarding probiotic supplementation during pregnancy. Because allocation was preference-based and no placebo was used, participants and clinical investigators were not blinded to group assignment. Laboratory personnel processing the vaginal cultures remained unaware of participants’ group allocation. The individual pregnant woman was both the unit of assignment and the unit of analysis. Participants in both groups were followed according to the same study schedule, with repeat vaginal GBS culture planned at 34–36 weeks of gestation. No enrolled participant received systemic antibiotic therapy between enrolment and the follow-up culture assessment.

Participants who chose the probiotic group received a prescription for Crispact^®^ orosoluble stick packs, a commercially available food supplement manufactured by Labomar S.p.A. (Istrana, Treviso, Italy) and marketed by PharmExtracta S.p.A. (Pontenure, Piacenza, Italy), and purchased the product independently from community pharmacies. The probiotic was not supplied free of charge by the investigators, the hospital, the manufacturer, or any study sponsor. Because participants purchased the product independently from different community pharmacies, batch/lot numbers and individual expiry dates were not recorded and could not be reliably retrieved retrospectively.

Each Crispact^®^ orosoluble stick was declared to contain at least 20 × 10^9^ colony-forming units (CFU) of *Lactobacillus crispatus* M247, with the declared count applying through the end of the stated shelf life when the product was stored as directed. Participants self-administered one stick orally at home once daily for eight weeks, beginning after confirmation of vaginal GBS colonization at 24–26 weeks of gestation. At enrollment, participants received standardized administration and storage instructions, including storage protected from light at 2–8 °C and transport at temperatures below 25 °C for no longer than 48 h. Treatment adherence was assessed through patient self-report during routine antenatal visits; no additional adherence-enhancement measures, such as reminder systems or sachet counts, were used. The control group received standard antenatal care without probiotic supplementation.

Vaginal specimens were collected during antenatal visits, from the lower third of the vagina using sterile swabs (DELTALAB S.L., Rubí, Spain) and transported promptly to the microbiology laboratory without a transport or preservation medium, in accordance with the hospital’s internal microbiology procedures. All specimens were processed in the same microbiology laboratory at Elias University Emergency Hospital, according to the Romanian national microbiology guideline and a uniform local protocol. All specimens were processed within 30 min of receipt in the laboratory. Samples were inoculated into a selective enrichment broth for GBS (Todd–Hewitt broth with CNA; Oxoid Deutschland GmbH, Wesel, Germany) and, following enrichment, subcultured onto Columbia agar supplemented with sheep blood (Oxoid Deutschland GmbH, Wesel, Germany) and a GBS-selective chromogenic agar (Brilliance GBS Agar; Oxoid Deutschland GmbH, Wesel, Germany). Cultures were incubated aerobically at 35–37 °C for 16–24 h. Presumptive GBS colonies were confirmed as *Streptococcus agalactiae* using matrix-assisted laser desorption/ionization time-of-flight mass spectrometry (MALDI-TOF MS) on an IVD MALDI Biotyper sirius system (Bruker Daltonics GmbH & Co. KG, Bremen, Germany), with software IVD-12299 Library Module version 5.2.320 (2023). Results were reported qualitatively as GBS present or absent. Rectal sampling was not included because the study protocol was designed to assess vaginal GBS colonization, rather than rectal or gastrointestinal carriage.

Concomitant *Candida* spp. or coccobacilli were incidental findings from the same screening vaginal specimen in asymptomatic participants. Coccobacilli were identified by optical microscopy (Leica DM100 LED microscope—Leica Microsystems CMS GmbH, Wetzlar, Germany), while the corresponding cultures were negative. No antibiotic or antifungal treatment was indicated or administered. Women requiring systemic antibiotic or intravaginal antimicrobial therapy were excluded before enrolment.

The primary outcome was persistent vaginal GBS colonization at follow-up, defined as a positive vaginal culture for *Streptococcus agalactiae* at 34–36 weeks of gestation. The study objectives are summarized in Table 2.

Safety assessment was exploratory. At each scheduled antenatal visit during the intervention period, participants in the probiotic group were verbally asked whether they had experienced any adverse events since the previous visit, using a yes/no question. No standardized or validated adverse-event questionnaire, prespecified symptom checklist, severity grading, or formal causality assessment was used. Control-group participants received routine antenatal care, including standard clinical questioning.

The absence of systemic antibiotic exposure between enrolment and follow-up was ascertained by questioning participants at scheduled antenatal visits.

The study was registered retrospectively in the ISRCTN registry (ISRCTN47071089). At study initiation, our approach to registration was based on the regulatory classification of the intervention in Romania as a commercially available food supplement rather than an investigational medicinal product. During the editorial process, following discussions with the Editorial Office regarding the international prospective trial-registration standards applicable to publication, we registered the study retrospectively in the ISRCTN registry, as requested, to ensure full transparency. Statistical analyses were performed using jamovi (version 2.7.30; The jamovi project, Sydney, Australia). Firth penalized logistic regression was conducted using the ClinicoPath module (version 1.0.0). Continuous variables were summarized as mean ± standard deviation (SD), whereas categorical variables were presented as absolute numbers and percentages. Baseline characteristics were compared between the probiotic and control groups to assess group comparability. Continuous variables were assessed for normality and compared using the independent samples *t*-test, while categorical variables and outcomes were analyzed using Fisher’s exact test because of the small sample size and expected cell counts. The primary outcome was assessed by calculating the relative risk (RR) of persistent vaginal GBS colonization together with 95% confidence intervals (95% CI). Relative risk reduction (RRR), absolute risk reduction (ARR), and number needed to treat (NNT) were also calculated to evaluate the clinical magnitude of the observed effect.

The 95% confidence interval for the absolute risk reduction was calculated using the Newcombe hybrid-score method, and the corresponding confidence interval for the number needed to treat was obtained by inverting the confidence limits of the absolute risk reduction. Given the limited number of outcome events and the presence of sparse cells, factors independently associated with persistent GBS colonization at follow-up were explored using Firth’s penalized logistic regression. Persistence of GBS colonization at the third-trimester assessment was entered as the outcome, while study group, maternal age, body mass index (BMI), and history of GBS colonization in a previous pregnancy were included as explanatory variables. Maternal age and BMI were entered as continuous variables. The primary exploratory model included all 52 participants with available follow-up data and 19 persistence events. Previous GBS colonization history was entered as a categorical variable with three levels (“no”, “yes”, and “unknown”), thereby retaining the eight participants with unknown previous colonization status. A sensitivity analysis was subsequently performed after excluding these eight participants; this analysis included 44 participants and 15 persistence events. Adjusted odds ratios (aORs) with 95% profile-likelihood confidence intervals and *p* values were reported. Given the limited number of events relative to the number of model parameters, both adjusted analyses were considered exploratory. The primary outcome was analyzed using a complete-case approach and included participants for whom the follow-up vaginal culture at 34–36 weeks was available. No imputation was performed for missing outcome data. One participant from the control group who did not attend the follow-up assessment was considered lost to follow-up and was not included in the primary outcome analysis. A two-sided *p*-value < 0.05 was considered statistically significant.

## 3. Results

During the recruitment period, 339 pregnant women underwent study-specific vaginal GBS testing at 24–26 weeks of gestation. Of these, 64 women (18.9%) had a positive vaginal GBS culture and were assessed for eligibility, whereas 275 had a negative culture. Nine GBS-positive women were excluded because they required systemic antibiotic treatment for other medical or obstetric conditions, and two eligible women declined participation. Consequently, 53 women provided written informed consent and were enrolled in the study. Following counselling, 24 women selected the probiotic group and 29 selected the control group. One participant from the control group did not attend the follow-up assessment at 34–36 weeks and was considered lost to follow-up. The final complete-case analysis therefore included 52 participants: 24 in the probiotic group and 28 in the control group. Participant flow is presented in Figure 1.

The baseline demographic and clinical characteristics of the study population were comparable between the probiotic and control groups.

Mean maternal age was 32.50 ± 3.53 years in the probiotic group and 32.50 ± 4.11 years in the control group (*p* = 1.00). Mean body mass index (BMI) was also similar between groups (29.18 ± 2.25 vs. 29.23 ± 2.04 kg/m^2^, *p* = 0.93). The proportion of smokers did not differ significantly (12.5% vs. 14.3%, *p* = 1.00), nor did the prevalence of maternal comorbidities (25.0% vs. 28.6%, *p* = 1.00). Parity distribution was comparable between the two groups (*p* = 1.00), with similar proportions of primiparous, secundiparous, and multiparous women. The prevalence of uterine scar was also similar (*p* = 0.69). Progesterone use during pregnancy did not differ between groups (12.5% vs. 14.3%, *p* = 1.00). Likewise, the history of previous GBS colonization and the distribution of associated vaginal infections detected at the second trimester (T2) screening were comparable between groups (*p* = 0.92 and *p* = 0.33, respectively). Nevertheless, *Candida* spp. was detected more frequently in the control group than in the probiotic group (5/28, 17.9% vs. 1/24, 4.2%, respectively). Given the small number of observations, this imbalance is reported descriptively. Overall, no statistically significant differences were observed in baseline characteristics between the groups (Table 3).

Baseline characteristics are presented for the complete-case analysis population; the participant lost to follow-up from the control group was not included. The control group included arterial hypertension alone (n = 3), diabetes mellitus alone (n = 3), coexisting hypertension and diabetes mellitus (n = 1), and another comorbidity (n = 1). The probiotic group included arterial hypertension alone (n = 2), diabetes mellitus alone (n = 2), coexisting arterial hypertension and diabetes mellitus (n = 1), and another comorbidity (n = 1). T2, second trimester assessment at 24–26 weeks of gestation.

In the control group, 16 out of 28 women (57.1%) remained GBS-positive at follow-up, whereas persistent colonization was observed in only 3 of 24 women (12.5%) in the probiotic group who received oral *Lactobacillus crispatus* M247 for eight weeks. Correspondingly, the follow-up vaginal culture was negative in 12 of 28 women (42.9%) in the control group and in 21 of 24 women (87.5%) in the probiotic group.

Comparative analysis using Fisher’s exact test demonstrated a statistically significant difference between the two groups (*p* = 0.001). The relative risk (RR) of persistent colonization in the probiotic group compared with the control group was 0.219 (95% CI 0.072–0.661), corresponding to a relative risk reduction (RRR) of 78.1%. The absolute risk reduction was 44.6% (95% CI 18.8–62.9%), corresponding to a number needed to treat of 2.24 (95% CI 1.59–5.33) to prevent one additional case of persistent detectable vaginal GBS colonization.

Self-reported treatment adherence in the probiotic group was 100%, and no adverse events were reported in response to the non-structured yes/no questioning. No deviations from the prescribed dosing regimen were reported in the probiotic group. The only departure from the planned study schedule was the missed follow-up visit by one participant in the control group.

An exploratory subgroup analysis was performed among women with a documented history of GBS colonization in previous pregnancies. At follow-up, all four women in the probiotic group had a negative vaginal GBS culture, whereas all four women in the control group had persistent detectable vaginal GBS colonization. Given the very small subgroup size and complete separation of outcomes, these findings are presented descriptively without inferential statistical testing.

A total of 19 persistence events were observed among the 52 participants included in the primary exploratory model. In the Firth penalized logistic regression analysis, probiotic supplementation was associated with lower odds of persistent GBS colonization after adjustment for maternal age, BMI, and previous GBS colonization status (aOR 0.13, 95% CI 0.03–0.46; *p* = 0.001). Maternal age, BMI, and previous GBS colonization status were not significantly associated with persistence (Table 4).

Eight participants had an unknown previous GBS colonization status and were retained as a separate category in the primary model. A sensitivity analysis excluding these participants included 44 women and 15 persistence events. The estimated association was modestly attenuated but remained statistically significant (aOR 0.17, 95% CI 0.03–0.63; *p* = 0.007). Thus, exclusion of participants with unknown previous colonization status did not materially alter the direction or interpretation of the association.

## 4. Discussion

In this prospective, non-randomized, contemporaneous controlled intervention study, oral administration of *Lactobacillus crispatus* M247 during the second trimester was associated with a significantly lower rate of persistent vaginal GBS colonization at 34–36 weeks of gestation compared with standard prenatal care.

Women receiving the probiotic regimen demonstrated a markedly higher proportion of negative follow-up vaginal cultures, with persistent colonization observed in only 12.5% of cases compared with 57.1% in the control group. This corresponded to a relative risk reduction of approximately 78.1%, supporting an association between probiotic supplementation and follow-up vaginal GBS culture status.

Beyond statistical significance, the observed effect size was clinically meaningful. The observed absolute risk reduction of 44.6% (95% CI 18.8–62.9%) corresponded to an unrounded number needed to treat of 2.24 (95% CI 1.59–5.33).

In the full-cohort exploratory Firth model, probiotic supplementation remained associated with lower odds of persistent GBS colonization after adjustment for maternal age, BMI, and previous GBS colonization status. The sensitivity analysis excluding participants with unknown previous colonization status produced an estimate with the same direction and a similar magnitude. However, because the number of outcome events was limited relative to the number of fitted parameters, these adjusted findings should be regarded as exploratory and hypothesis-generating rather than as evidence of an independent causal effect.

GBS colonization remains a major obstetric concern because of its association with early-onset neonatal sepsis. Current international guidelines therefore recommend universal screening in late pregnancy followed by intrapartum antibiotic prophylaxis for colonized women as the most effective strategy for preventing neonatal disease [2,3]. However, this approach does not eradicate maternal colonization before delivery and does not address the ecological imbalance of the vaginal microbiota that may predispose to GBS persistence. For this reason, increasing attention has been directed toward microbiota-based interventions, including the use of probiotics during pregnancy. In our study, the potential confounding effect of systemic antibiotic exposure was minimized because women receiving antibiotics during the study period were excluded.

Several studies have explored the potential role of probiotic supplementation in reducing GBS colonization, although the available evidence remains heterogeneous. Randomized and pilot clinical trials investigating oral probiotic administration during pregnancy have produced mixed results, with some studies suggesting feasibility and possible reductions in GBS carriage, while others did not demonstrate significant differences compared with placebo [8,9]. A pilot study involving pregnant women with vaginal GBS colonization evaluated the administration of a multi-strain lactic acid bacteria preparation, including *Lactobacillus crispatus*, given orally (2 × 2 g/day) starting in the second trimester and continued for 19 weeks. By 34–35 weeks of gestation, approximately 90% of the participants tested negative for GBS colonization [8]. In contrast, studies that did not demonstrate a significant reduction in GBS colonization generally initiated probiotic supplementation later in pregnancy, typically during the third trimester (after at least 33 weeks of gestation) [9,10,11].

The vaginal microbiota plays an important role in pregnancy outcomes, with vaginal communities dominated by *Lactobacillus crispatus* consistently associated with a lower risk of preterm birth. In this context, live vaginal biotherapeutics containing *L. crispatus* have been investigated as a strategy to modulate the vaginal microbiome during pregnancy. An observational study evaluating *Lactobacillus crispatus* CTV-05 (LACTIN-V) administered from 14 weeks of gestation demonstrated good safety, tolerability, and high patient acceptability, with no serious adverse events reported. Although the study was not designed to assess clinical efficacy, the findings support the feasibility of microbiome-targeted interventions during pregnancy and highlight the need for further trials to evaluate their potential impact on obstetric outcomes [12].

A large study analyzing 1860 vaginal swabs from women in the third trimester found that the presence of lactobacilli (particularly *Lactobacillus crispatus*) was significantly inversely associated with GBS colonization, suggesting that a lactobacilli-dominated vaginal microbiota may help protect against GBS carriage [13]. A randomized, double-blind, placebo-controlled trial evaluating oral probiotic supplementation for the secondary prevention of vulvovaginal infections (abnormal vaginal flora, bacterial vaginosis, vulvovaginal candidiasis) during pregnancy found no significant reduction in infection recurrence compared with placebo. Despite prolonged administration throughout pregnancy, the probiotic strains (*Bifidobacterium bifidum*, *Bifidobacterium lactis*, *Lactobacillus* (L.) *acidophilus*, *L. paracasei*, *L. rhamnosus* and *Streptococcus thermophilus*: >6 × 10^9^ CFU/capsule) did not establish detectable vaginal colonization, and pregnancy outcomes were similar between the two groups [14].

A randomized placebo-controlled trial evaluating oral probiotic supplementation with *Lactobacillus rhamnosus* GR-1 and *Lactobacillus reuteri* RC-14 during pregnancy (for 12 weeks at 23–25 weeks of gestation) found that probiotic administration was feasible and well tolerated, with high treatment compliance. However, the intervention did not significantly reduce the rate of recto-vaginal GBS colonization at 35–37 weeks of gestation compared with placebo [15].

From a biological perspective, the potential benefit of probiotic administration is supported by the central role of vaginal microbiota composition in maintaining mucosal homeostasis during pregnancy. Vaginal microbial communities dominated by *Lactobacillus* species, particularly *Lactobacillus crispatus*, are generally associated with a stable, low-pH environment that inhibits the growth of pathogenic organisms [16]. Several microbiome studies have demonstrated an inverse relationship between the presence of *Lactobacillus*, especially *L. crispatus*, and GBS colonization in pregnant women [5].

Moreover, *L. crispatus*-dominated microbiota profiles have also been linked to favorable obstetric outcomes, including a lower risk of preterm birth, suggesting that restoration or maintenance of a lactobacilli-dominant vaginal ecosystem may represent a protective factor in pregnancy [6].

Comparative evidence concerning oral versus vaginal probiotic administration is derived primarily from studies of bacterial vaginosis rather than GBS colonization [17]. To our knowledge, no clinical trials have specifically evaluated vaginal probiotic administration for reducing maternal GBS colonization. Therefore, no conclusions can currently be drawn regarding the relative efficacy of oral and vaginal administration for this indication, and this represents an important gap in the literature. In clinical practice, oral administration may offer greater acceptability and adherence, which could be particularly relevant in pregnant populations.

GBS colonization is dynamic and may be intermittent. Because only one vaginal specimen was collected at each study time point, a negative follow-up culture cannot be distinguished with certainty from intermittent carriage, bacterial levels below the detection threshold, or sampling variability. This consideration is particularly relevant because 42.9% of untreated controls also had a negative follow-up culture. Accordingly, the observed outcome should be interpreted as the absence of detectable vaginal GBS colonization at the time of follow-up rather than as confirmed bacterial clearance or eradication. Because rectal sampling was not performed, a negative follow-up vaginal culture could not be interpreted as absence or eradication of rectal or gastrointestinal carriage, and subsequent vaginal recolonization remained possible. Future studies should incorporate serial, study-specific vaginal cultures, combined vaginal-rectal screening, and, where feasible, sampling at delivery.

Taken together, the available literature provides a biological rationale for investigating probiotic supplementation as a potential adjunctive strategy for maternal GBS colonization. However, the present findings concern vaginal GBS culture status and do not demonstrate modulation of the vaginal microbiota. Current evidence remains insufficient to replace established screening and intrapartum prophylaxis protocols. Future research should focus on identifying optimal probiotic strains, treatment timing, and administration routes, as well as clarifying whether microbiome-targeted interventions can meaningfully reduce maternal colonization and improve neonatal outcomes.

In the exploratory subgroup of women with a documented history of GBS colonization, negative follow-up vaginal cultures were observed in all four participants receiving probiotic supplementation, whereas persistent detectable vaginal colonization was observed in all four controls. Because this subgroup comprised only eight participants and showed complete separation of outcomes, no inferential conclusions can be drawn. This descriptive observation should be regarded as hypothesis-generating and requires confirmation in larger, adequately powered studies with prespecified subgroup analyses.

The present study has several strengths. First, the prospective design allowed for a clearly defined temporal relationship between probiotic administration and microbiological follow-up. Second, the study focused specifically on pregnant women with confirmed GBS colonization during the second trimester, ensuring a well-defined study population.

In addition, follow-up microbiological assessment was available for 52 of the 53 enrolled participants (98.1%). Only one participant from the control group was lost to follow-up, resulting in minimal post-enrolment attrition.

Several limitations should be acknowledged. This single-center study included a relatively small number of participants, which may limit the generalizability of the findings. Group allocation was not randomized and was based on patient preference, which may have introduced selection bias. Women who chose probiotic supplementation may have differed from those who chose standard care in unmeasured characteristics, including previous probiotic exposure, concurrent use of other dietary supplements, dietary habits, health-seeking behaviour, educational attainment, socioeconomic factors, and attitudes toward complementary or microbiota-directed interventions. These variables were not prospectively collected and therefore could not be incorporated into the adjusted analyses. Consequently, the observed association should not be interpreted as establishing a causal effect of probiotic supplementation.

Vaginal pH measurements, comprehensive microbiota profiling, and strain-specific testing for vaginal colonization by *L. crispatus* M247 were not performed. Therefore, the study cannot establish whether supplementation modified the vaginal microbiota or whether such changes mediated the observed association with vaginal GBS persistence. Potential effects on the vaginal microbiota may vary between individuals, and this variability could not be characterized in the present study.

Neonatal outcomes were not evaluated in the present study.

Because participants purchased the product independently from different community pharmacies, batch and lot numbers and individual expiry dates were not recorded. Consequently, batch-specific product characteristics and probiotic viability at the time of administration could not be independently verified.

Routine combined vaginal-rectal screening results and delivery specimens were not prospectively collected as study variables and could not be retrieved for the present analysis.

In addition, the investigators who provided counselling, enrolled participants, and collected vaginal specimens were not blinded to group allocation, which may have introduced the potential for observer bias.

The absence of a transport medium could theoretically have reduced culture sensitivity; however, because the same sampling and processing protocol was applied in both groups, any resulting misclassification would be expected to be non-differential.

The adjusted analyses were also limited by the small number of persistence events relative to the number of fitted parameters. Although Firth penalization was used to reduce small-sample bias arising from sparse data, it cannot eliminate model instability, imprecision, or residual confounding. Accordingly, the multivariable analyses were considered exploratory. Unknown previous GBS colonization status was retained as a separate category in the full-cohort model. Its exclusion in the sensitivity analysis modestly attenuated the probiotic estimate from 0.13 to 0.17 without changing the direction of the association. No formal sample size calculation was performed because this was an exploratory pilot study. Finally, the study focused on antenatal GBS persistence and did not evaluate intrapartum colonization or neonatal outcomes.

An additional potential source of residual confounding is the observed baseline imbalance in the vaginal microbial environment. *Candida* spp. was detected in 17.9% of controls and 4.2% of participants in the probiotic group. Although the overall distribution of second-trimester vaginal infection categories did not differ statistically between groups, this imbalance may reflect differences in the underlying vaginal flora that could plausibly be associated with subsequent GBS persistence. Given the limited number of persistence events and the small number of participants with *Candida* detection, this variable was not included in the adjusted model. The direction and magnitude of any resulting confounding remain uncertain.

Because the primary outcome was analysed using a complete-case approach, attrition bias cannot be entirely excluded. However, only one of the 53 enrolled participants was lost to follow-up, and the missing outcome occurred in the control group. Because only one participant was lost to follow-up, a formal comparison of baseline characteristics between participants retained in the analysis and the participant lost to follow-up was not considered meaningful and was not performed.

Adherence was self-reported and was not objectively verified by sachet count; therefore, incomplete intake cannot be entirely excluded. Complete self-reported adherence in this small, preference-allocated cohort may not be generalizable to routine clinical practice.

Adverse-event ascertainment relied on non-structured questioning at scheduled antenatal visits, with the possibility of underreporting. The absence of reported adverse events should therefore be interpreted within the limits of this assessment.

Despite these limitations, oral *Lactobacillus crispatus* M247 supplementation was associated with a lower frequency of persistent detectable vaginal GBS colonization. The consistency of the association across the two exploratory Firth models supports further evaluation in adequately powered randomized trials but does not establish efficacy or causality.

Further research is required to confirm these findings in larger randomized controlled trials. Future studies should also determine whether probiotic supplementation reduces intrapartum GBS colonization and neonatal exposure, and whether any observed effects are mediated by changes in the vaginal microbiota.

## 5. Conclusions

Oral *Lactobacillus crispatus* M247 supplementation from 24–26 weeks of gestation was associated with a lower frequency of detectable vaginal GBS colonization at 34–36 weeks in this small, preference-allocated cohort. Self-reported adherence was complete, and no adverse events were reported among participants receiving the probiotic. These findings are hypothesis-generating and larger randomized controlled trials with longitudinal microbiological assessment are needed to confirm these findings.

## Figures and Tables

**Figure 1:**
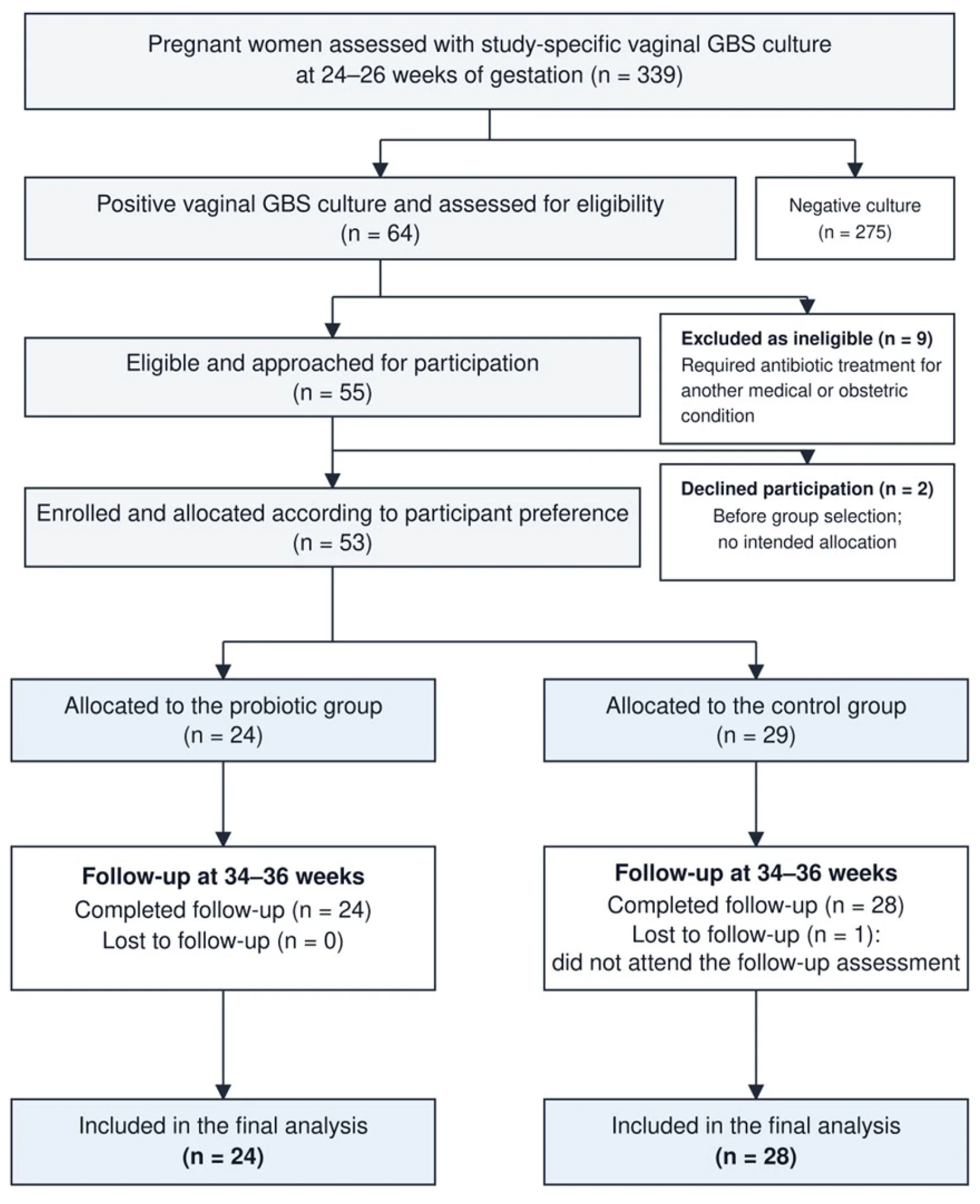
Flow diagram of participant screening, enrolment, group allocation, follow-up, and final analysis. GBS, Group B Streptococcus. Abbreviations: n: number; GBS: Group B Streptococcus.

**Table 1:** Inclusion and exclusion criteria.

Inclusion Criteria	Exclusion Criteria
Pregnant women aged ≥18 years Confirmed viable pregnancy Vaginal GBS-positive at 24–26 weeks of gestation Informed consent	Use of systemic antibiotics Concomitant probiotic use Intravaginal antimicrobials Major immunosuppression

**Table 2:** Objectives of the study.

Primary Objective	Secondary Objectives
To evaluate whether oral administration of *Lactobacillus crispatus* M247 during the second trimester is associated with lower persistence of vaginal GBS colonization at 34–36 weeks of gestation compared with standard prenatal care.	- To compare the proportions of participants with a negative follow-up vaginal GBS culture between the probiotic and control groups. - To describe self-reported treatment adherence and maternal adverse events reported during oral *Lactobacillus crispatus* M247 supplementation. - To perform exploratory subgroup analyses according to baseline clinical characteristics, including previous GBS colonization history.

**Table 3:** Baseline characteristics.

Variable	Probiotic (n = 24)	Control (n = 28)	*p*-Value
Age (years)	32.50 ± 3.53	32.50 ± 4.11	1.00
BMI	29.18 ± 2.25	29.23 ± 2.04	0.93
Smokers	3 (12.5%)	4 (14.3%)	1.00
Comorbidities	6 (25.0%)	8 (28.6%)	1.00
Parity (overall)			1.00
• Primiparous	10 (41.7%)	11 (39.3%)	
• Secundiparous	8 (33.3%)	10 (35.7%)	
• Multiparous	6 (25.0%)	7 (25.0%)	
Uterine scar (overall)			0.69
• No scar	15 (62.5%)	15 (53.6%)	
• 1 scar	8 (33.3%)	10 (35.7%)	
• 2 scars	1 (4.2%)	3 (10.7%)	
Progesterone use	3 (12.5%)	4 (14.3%)	1.00
Previous GBS (overall)			0.92
• No	17 (70.8%)	19 (67.9%)	
• Yes	4 (16.7%)	4 (14.3%)	
• Unknown	3 (12.5%)	5 (17.9%)	
Microbiological findings at T2			0.33
• Absent	21 (87.5%)	21 (75.0%)	
• *Candida* spp.	1 (4.2%)	5 (17.9%)	
• Coccobacilli	2 (8.3%)	2 (7.1%)	

**Table 4:** Exploratory Firth penalized logistic regression analyses of persistent vaginal GBS colonization at follow-up.

Variable	Full Cohort, n = 52; 19 Events, aOR (95% CI)	*p* Value	Sensitivity Analysis, n = 44; 15 Events, aOR (95% CI)	*p* Value
Probiotic group vs. control	0.13 (0.03–0.46)	0.001	0.17 (0.03–0.63)	0.007
Maternal age, per year	1.06 (0.89–1.27)	0.492	1.05 (0.88–1.27)	0.574
BMI, per kg/m^2^	1.07 (0.79–1.46)	0.672	1.11 (0.79–1.63)	0.548
Previous GBS colonization: yes vs. no	2.79 (0.53–16.49)	0.222	2.57 (0.52–14.03)	0.241
Previous GBS colonization: unknown vs. no	1.53 (0.24–9.63)	0.644	—	—

Abbreviations: aOR, adjusted odds ratio; BMI, body mass index; CI, confidence interval; GBS, Group B Streptococcus. Values are adjusted odds ratios with 95% profile-likelihood confidence intervals. The reference categories were the control group and no previous GBS colonization. The full-cohort model retained unknown previous GBS colonization status as a separate category. The sensitivity analysis excluded the eight participants with unknown previous colonization status. Both models used Firth’s penalized likelihood method and should be interpreted as exploratory because of the limited number of outcome events.

## Data Availability

The data supporting the findings of this study are available from the corresponding author upon reasonable request. The data are not publicly available due to privacy and ethical restrictions involving human participants.

## Supplementary Materials

The following supporting information can be downloaded at: https://www.mdpi.com/article/10.3390/jcm15197368/s1, Table S1: TREND Statement Checklist.
